# Genetic correlation between genes targeted by lipid-lowering drugs and venous thromboembolism: A drug-target Mendelian randomization study

**DOI:** 10.1097/MD.0000000000040770

**Published:** 2024-12-20

**Authors:** Min Li, Hangyu Duan, Jinwen Luo, Yu Tan, Min Liu, Xiaohan Zhao, Dazhuo Shi, Xiaojuan Ma

**Affiliations:** a Xiyuan Hospital, China Academy of Chinese Medical Sciences, Beijing, China; b National Clinical Research Center for Chinese Medicine Cardiology, Xiyuan Hospital, China Academy of Chinese Medical Sciences, Beijing, China.

**Keywords:** deep vein thrombosis, lipid-lowering drug, Mendelian randomization of drug targets, pulmonary embolism, venous thromboembolism

## Abstract

Dyslipidemia has been established as a potential risk factor for venous thromboembolism (VTE) in several observational studies. Statins and novel lipid-modifying agents are being explored for their potential in VTE prevention, encompassing deep vein thrombosis (DVT), and pulmonary embolism (PE). Nonetheless, conclusive evidence supporting the effectiveness remains uncertain. Without definitive proof, the current recommendation of lipid-lowering drugs (LLDs) for preventing VTE, either primarily or secondarily, is not support. An investigation into the impact of 8 classes of LLDs on VTE was conducted using a drug-target Mendelian randomization approach. The drug categories examined included 3-hydroxy-3-methylglutaryl-CoA reductase (HMGCR), apolipoprotein B, proprotein convertase subtilisin/kexin type 9, Niemann–Pick C1-like 1, lipoprotein lipase (LPL), angiopoietin-like 3, apolipoprotein C3 (APOC3), and peroxisome proliferator-activated receptor alpha. Leveraging genetic variants situated proximate to or within drug-target genes linked with low-density lipoprotein and triglycerides, we acted as proxies for LLDs. The UK Biobank study was the source of data on VTE, PE, and DVT of lower extremities (LEDVT). We employed the inverse-variance weighted method for the core analysis in Mendelian randomization, complemented by sensitivity analysis to investigate horizontal pleiotropy and heterogeneity. Employing genetic proxies to inhibit HMGCR revealed a notable correlation with reduced LEDVT risk (odds ratio [OR]: 0.995, 95% CI: 0.992–0.998, *P* = .002), VTE (OR: 0.994, 95% CI: 0.988–1.000, *P* = .033), but a no significant association with PE (OR: 1.000, 95% CI: 0.994–1.002, *P* = .246). The suppression of APOB was linked with an elevated risk of experiencing LEDVT (OR: 1.002, 95% CI: 1.001–1.004, *P* = .006), VTE (OR: 1.005, 95% CI: 1.002–1.007, *P* < .001), and PE (OR: 1.002, 95% CI: 1.000–1.004, *P* = .031). Similarly, the activation of LPL was associated with increased risks for VTE (OR: 1.003, 95% CI: 1.001–1.005, *P* = .003) and PE (OR: 1.003, 95% CI: 1.002–1.005, *P* < .001). Additionally, the inhibition of APOC3 was linked to a higher DVT risk (OR: 1.002, 95% CI: 1.000–1.004, *P* = .038). Research has shown that HMGCR, out of 8 lipid-lowering drug-targets evaluated, exhibited a significant correlation with VTE and LEDVT, highlighting its potential as an effective target for the treatment or prevention of these conditions. In contrast, APOB, LPL, and APOC3 each contribute to an increased risk of VTE, PE, and LEDVT in various degrees, pharmacovigilance for VTE, PE, and LEDVT risk among users of APOB inhibitors, LPL activation, and APOC3 inhibitors may be warranted.

## 1. Introduction

Venous thromboembolism (VTE), which is comprised of deep vein thrombosis (DVT) and pulmonary embolism (PE), ranks among the top 5 most prevalent vascular conditions worldwide.^[1]^ The American Heart Association’s 2021 report estimates that there are approximately 1220,000 new VTE cases every year in the United States alone.^[2]^ VTE stands as a significant mortality factor in vascular diseases, evidenced by a study from the RIETE registry, which included around 121,190 patients from 26 nations, revealing 30-day mortality rates of 2.55% for DVT in the lower limbs and 5.05% for pulmonary embolism.^[3]^ Agents for pharmacological thromboprophylaxis, such as warfarin, low-molecular weight heparin, and new oral anticoagulants, demonstrate efficacy in VTE prevention, they also pose an elevated risk of bleeding.^[4]^ Despite significant strides in understanding VTE epidemiology and the presence of effective primary and secondary precautions, its incidence has not diminished over recent decades.^[5–7]^ Recent evidence indicates a positive link between traditional atherosclerosis risk factors and VTE. Circulating lipids exhibit both prothrombotic and endothelium-damaging effects. Analyzing thirty-three case-control studies with 185,124 participants revealed that patients with VTE had significantly higher levels of total cholesterol and triglyceride, and significantly lower levels of high-density lipoprotein cholesterol compared to those without VTE.^[8]^ Another study found a slight but significant correlation between lipoprotein(a) (Lp(a)) and a heightened risk of VTE.^[9]^ Statins, which are 3-hydroxy-3-methyglutaryl-coenzyme A reductase inhibitors, are widely known for their ability to prevent cardiovascular diseases by lowering lipids.^[10,11]^ Recent findings suggest that statins might also offer protection against VTE.^[12,13]^ Furthermore, emerging lipid-modifying agents like proprotein convertase subtilisin/kexin type 9 (PCSK9) have shown promise in reducing VTE risk, although the evidence remains uncertain.^[14,15]^

Considered the pinnacle for establishing drug treatment causality, Randomized Controlled Trials are often hindered by their costliness and various practical challenges, While Mendelian randomization (MR) analysis emerges as a viable alternative. By employing genetic variants as tools for determining causality between exposure and outcome, MR analysis can verify if the observed associations are consistent with a causal effect.^[16]^ The evolution of fundamental theories and the proliferation of practical applications have made drug-target MR analysis a key method for exploring the effects of various agents, including inhibitors, agonists, antagonists, or activators on diseases and identifying potential therapeutic targets.^[17]^ This method led Steven Zhao and his team to identify an inverse relationship between PCSK9 inhibitors, as genetically proxied, and Psoriasis.^[18]^ In a study with a comparable design, Li and associates pinpointed lipoprotein lipase (LPL) as a promising therapeutic target for nonalcoholic fatty liver disease.^[19]^

Hence, our investigation utilized a drug-target MR approach to assess the sophisticated connection between lipid-lowering drug (LLDs) and VTE (comprising DVT and PE).

## 2. Methods

### 2.1. Study design

**Figure 1.**
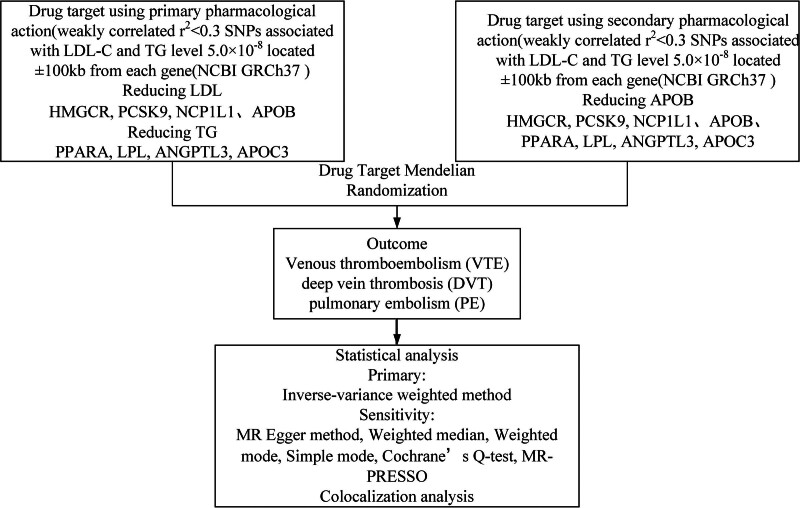
Summary of research methodology.

The influence of genetic variations on drug-target expression and function is profound, allowing predictions about drug effects based on genetic differences in the protein-targeting genes. This study’s methodology is graphically detailed in Figure 1 for a thorough overview. The analysis of anonymized, publicly accessible aggregated statistical data does not require additional ethical approval; therefore, the requirement for informed consent is waived.

### 2.2. Genetic proxies for lipid-lowering drug

Widely used LLDs along with innovative therapeutic agents were chosen in accordance with the latest dyslipidemia management guidelines.^[20,21]^ Comprehensive details regarding these datasets are outlined in Table S1, Supplemental Digital Content, http://links.lww.com/MD/O85. Subsequently, the DrugBank database was utilized to identify genes that encode the therapeutic targets of these drugs (https://www.drugbank.com/). Information regarding chromosomal locations and gene loci associated with the lipid-lowering medications were sourced from the National Center for Biotechnology Information gene database (https://www.ncbi.nlm.nih.gov/gene). We subdivided the target genes according to their chief pharmacological functions. Included in this classification are genes like HMGCR (3-hydroxy-3-methylglutaryl-CoA reductase), APOB (apolipoprotein B), PCSK9, and NPC1L1 (Niemann–Pick C1 like intracellular cholesterol transporter 1), which are key in lowering low-density lipoprotein C (LDL-C) levels. Additionally, PPARA (peroxisome proliferator-activated receptor alpha), LPL, ANGPTL3 (angiopoietin-like 3), and APOC3 (apolipoprotein C3) are identified for their role in the reduction of triglycerides (TG) levels.^[22]^ Genetic correlations with LDL and TG levels were derived from the most extensive genome-wide association study (GWAS) to data, conducted by the Global Lipids Genetics Consortium, with participation from around 1.3 million individuals of European descent.^[23]^

We identified single nucleotide polymorphisms (SNPs) associated with key lipid properties at a significant genome-wide threshold (*P* < 5.0 × 10^‐8^) to mimic the lipid-modifying influence of chosen drug targets. For this purpose, we used summary data from GWAS on LDL-C (for HMGCR, APOB, PCSK9, and NPC1L1), and TG (for PPARA, LPL, ANGPTL3, and APOC3), allowing us to pinpoint the genetic instruments for these drugs. To maximize the IV intensity of each LLD target gene, weak linkage disequilibrium (*r*^2^ < 0.3, window size = 10,000 kb) was permitted among SNPs.^[24]^ Since no genetic variants were identified in PPARA during the screening process, it was omitted from subsequent analysis.^[25]^ Details on the 7 drug targets that made it to the final analysis—HMGCR, APOB, PCSK9, NPC1L1, LPL, ANGPTL3, and APOC3, could be found in Table 1.

**Table 1:** Types of lipid-lowering drugs, compounds, and gene targets.

Primary lipid modulation	Gene	Chromosome	Base pair (GRCh37)	Drug-target	Mechanism of action	SNP	Examples of drugs
LDL-C (decreasing)	HMGCR	5	74632993–74657941	HMG-CoA reductase	HMGCR inhibition	7	Statins
	PCSK9	1	55505221–55530525	Proprotein convertase subtilisin/kexin type 9	PCSK9 inhibition	12	Evolocumab, alirocumab
	APOB	2	21224301–21266945	Apolipoprotein B-100	APOB inhibition	20	Mipomersen
	NPC1L1	7	44552134–44580929	Niemann–Pick C1-like 1 protein	NPC1L1 inhibition	3	Ezetimibe
TG (decreasing)	PPARA	22	46546429–46639653	Peroxisome proliferator-activated receptor alpha	PPARA enhancement	0	Fibrates
	LPL	8	19796764–19824770	Lipoprotein lipase	LPL activation	24	Ibrolipim (investigational)
	ANGPTL3	1	63063191–63071984	Angiopoietin-like 3	ANGPTL3 inhibition	4	Evinacumab (investigational)
	APOC3	11	116700623–116703788	Apolipoprotein C3	APOC3 inhibition	10	Volanesorsen (investigational)

LDL-C = low-density lipoprotein C, SNP = single nucleotide polymorphism, TG = triglycerides.

For confirming the robustness and consistency of our research outcomes, additional tests were performed by creating a different set of genetic tools. Apolipoprotein B (Apo-B), essential for the formation of LDL-C and TG, was employed to formulate genetic instruments targeting HMGCR, APOB, PCSK9, NPC1L1, LPL, ANGPTL3, and APOC3.^[26]^ Detailed information was provided in Table S3, Supplemental Digital Content, http://links.lww.com/MD/O85.

### 2.3. Outcome data

The main outcome measured was VTE, with secondary outcomes including left-sided deep vein thrombosis (LEDVT) and PE. Summary statistics from the UK Biobank, representing individuals of European ancestry,^[27]^ including data on VTE (4620 cases, 356,574 controls), PE (2118 cases, 359,076 controls) and LEDVT (2116 cases, 359,078 controls). The UK Biobank, a substantial prospective cohort study, captured over half a million people aged 40 to 69 from 2006 to 2010, maintaining a sustained interest in their well-being.^[28]^ To validate the suitability of genetic variants as targets for drug intervention, coronary heart disease (CHD) was employed as a validation measure, given the well-established correlation between lipid-lowering treatment and diminished CHD prevalence. CHD summary statistics, involving 60,801 cases and 123,504 controls, were obtained from the CARDIoGRAMplusC4D consortium.^[29]^

### 2.4. Statistical analysis

The inverse-variance weighted approach was commonly used to assess the causal impact of genetically mediated lipid-lowering targets on VTE, PE, and LEDVT, in the primary analysis of MR. For instruments with 2 or fewer genetic variants, we applied the Wald ratio for estimates. To ensure a valid association of variants with the lipid-lowering target, *F* statistics were computed by squaring β coefficient and dividing by the square of its standard error, with values over 10 indicating strong instrument reliability.^[30]^ For increased validity of our MR outcomes, we incorporated 4 extra sensitivity analysis techniques: MR-Egger,^[31]^ weighted median method,^[32]^ simple mode method, and weighted mode method.^[33]^ The existence of heterogeneity was gauged by employing Cochrane *Q*-test, with a *P*-value of <.05 serving as evidence of its presence.^[34]^ The assessment of possible horizontal pleiotropy in SNP was conducted via MR-Egger regression and Mendelian Randomization Pleiotropy RESidual Sun and Outlier analysis.^[35]^ The intercept in MR-Egger regression served as a signal for directional horizontal pleiotropy, as evidenced by a *P*-value under 0.05. The Mendelian Randomization Pleiotropy RESidual Sun and Outlier analysis was adept at identifying outlier data points potentially caused by horizontal pleiotropy. All statistical analyses were executed in R software version 4.2.3.

Furthermore, leave-one-out analyses were carried out to explore the influence of removing SNPs one by one from the instruments on the aggregate estimates of causality.^[36]^ To mitigate potential confounding arising from linkage disequilibrium, where variants closely linked to the authentic causal variant could influence the outcome through pathways unrelated to lipids, Bayesian colocalization analysis was carried out on drug targets significantly associated with the outcome.^[37]^ The analysis was conducted to investigate genetic confounding possibilities through the examination of posterior probabilities of diverse causal variants, the chances of shared causal variants existence, and the colocalization likelihood in cases involving a causal variant for the outcome.^[38]^ For assessing variants within the target genomic area’s connection with the exposure trait, outcome trait, or both, Bayesian colocalization employed standard prior probabilities of 10^‐4^, 10^‐4^, and 10^‐5^, respectively. The investigation assessed, through colocalization analysis, the likelihood (PP.H4) that specific genetic variations (SNPs) linked to drug targets and various conditions such as VTE, PE, and LEDVT could be traced back to the same causal variant at a specific locus. It also explored the chance (PP.H3) that separate causal variants, which are linked through linkage disequilibrium, might influence the drug targets and conditions separately. Drug targets demonstrating robust colocalization with VTE, PE, and LEDVT (PP.H4 > 0.75) were deemed as promising candidate genes.^[19]^ This research relied solely on anonymized summary data from previous studies, which all had the necessary ethical approvals and participant consent.

## 3. Results

Our research identified 7 SNPs within HMGCR, 12 in PCSK9, 3 in NPC1L1, 20 in APOB, 4 in ANGPTL3, 10 in APOC3, 24 in LPL as effective genetic tools. The strength of these tools was confirmed as their *F* statistics were well above the minimum acceptable value of 10, signifying their robustness (refer to Table S2, Supplemental Digital Content, http://links.lww.com/MD/O85 for further information). Within the framework of our positive control assessment, we detected noteworthy correlations between genetically proxied drug targets and a decrease in the risk of coronary heart disease (*P* < .05). This efficacy aligns with findings from prior research, underscoring the reliability of the genetic instruments^[39,40]^ (Figure S1, Supplemental Digital Content, http://links.lww.com/MD/O86 and Tables S10 and S11, Supplemental Digital Content, http://links.lww.com/MD/O85, http://links.lww.com/MD/O85).

**Figure 2.**
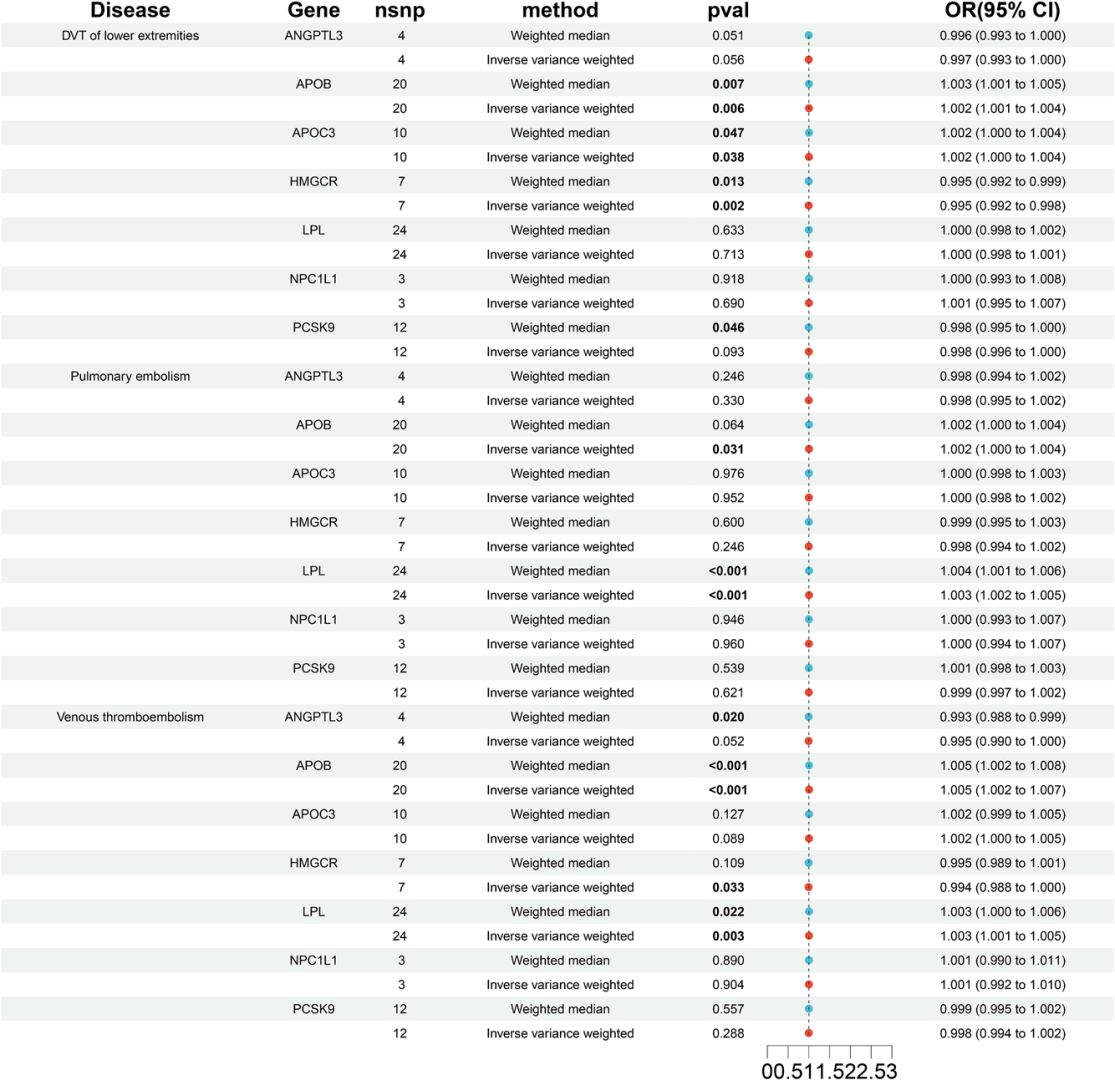
The relationship between genetically proxied drug targets and the peril of DVT, PE, and VTE via their lipid-modifying effects. DVT = deep vein thrombosis, PE = pulmonary embolism, VTE = venous thromboembolism.

Figure 2 and Tables S4–S6, Supplemental Digital Content, http://links.lww.com/MD/O85, http://links.lww.com/MD/O85, http://links.lww.com/MD/O85 presented the links between 7 categories of lipid-lowering medications and the risk of VTE, DVT and PE, respectively. Figures S2–S7, Supplemental Digital Content, http://links.lww.com/MD/O86, http://links.lww.com/MD/O86, http://links.lww.com/MD/O86, http://links.lww.com/MD/O86, http://links.lww.com/MD/O86, http://links.lww.com/MD/O86 showed scatter plots depicting these medications’ impact on susceptibility to VTE, DVT, and PE. Inhibition of HMGCR, which equates to a one standard deviation reduction in LDL-C levels, was notably connected with a decrease in the likelihood of developing VTE (OR: 0.994, 95% CI: 0.988–1.000, *P* = .033) and DVT (OR: 0.995, 95% CI: 0.992–0.998, *P* = .002). Conversely, inhibition of APOB, representative of a one standard deviation decrease in LDL, was found to significantly elevate the risk of VTE (OR: 1.005, 95% CI: 1.002–1.007, *P* < .001), DVT (OR: 1.002, 95% CI: 1.001–1.004, *P* = .006), and PE (OR: 1.002, 95% CI: 1.000–1.004, *P* = .031). Similarly, LPL activation increases the risk of VTE (OR: 1.003, 95% CI: 1.001–1.005, *P* = .003) and PE (OR: 1.003, 95% CI: 1.002–1.005, *P* = 9.79E‐06). APOC3 inhibition increases the risk of DVT (OR: 1.002, 95% CI: 1.000–1.004, *P* = .038).

No notable correlations were found between changes in primary lipid levels, as influenced by genetics involving ANGPTL3, NPC1L1, and PCSK9, and the occurrence of VTE, DVT, and PE.

**Figure 3.**
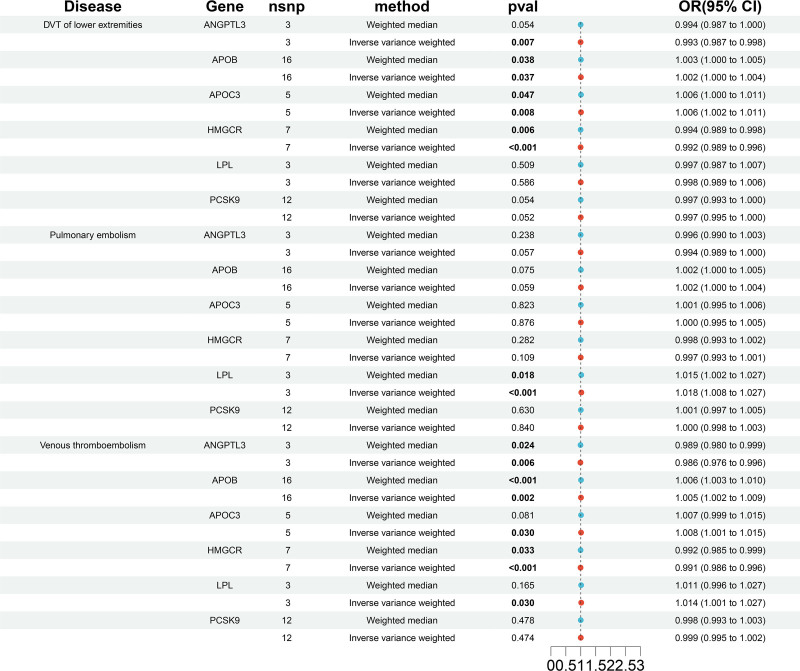
The relationship of genetically proxied drug targets with risk of DVT, PE, and VTE using alternative lipid-modifying effect. DVT = deep vein thrombosis, PE = pulmonary embolism, VTE = venous thromboembolism.

Simulating the gene effects weighted on Apo-B garnered similar outcomes (refer to Fig. 3, Tables S7–S9, Supplemental Digital Content, http://links.lww.com/MD/O85, http://links.lww.com/MD/O85, http://links.lww.com/MD/O85), although mimicking the inhibition of ANGPTL3 equivalent to a one standard deviation drop in Apo-B correlated with a decreased risk of VTE, DVT, and PE. Furthermore, the number of available instruments for genetic mimicking of NPC1L1 inhibition using Apo-B levels in relation to VTE, DVT, and PE are limited.

**Figure 4.**
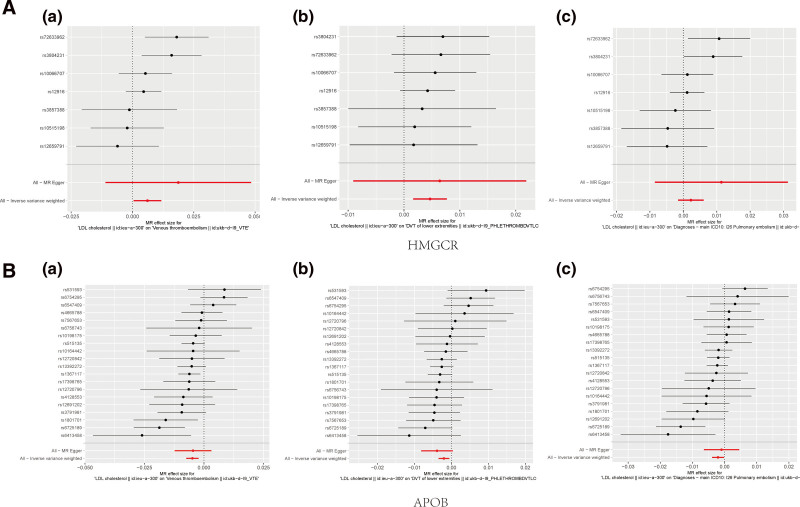
Forest plots depicted the estimated casual effects of each SNP within the HMGCR and APOB inhibition tools regarding VTE, DVT and PE. (A): (a) HMGCR inhibitor on VTE risk; (b) HMGCR inhibitor on DVT risk; (c) HMGCR inhibitor on PE risk. (B): (a) APOB inhibitor on VTE risk; (b) APOB inhibitor on DVT risk; (c) APOB inhibitor on PE risk. APOB = apolipoprotein B, DVT = deep vein thrombosis, HMGCR = 3-hydroxy-3-methylglutaryl-CoA reductase, PE = pulmonary embolism, SNP = single nucleotide polymorphism, VTE = venous thromboembolism.

Forest plots detailed the causal analysis of each SNP’s impact within HMGCR and APOB inhibitory tools on VTE, DVT, and PE risk (Fig. 4). Visualization of forest plots for additional drug targets were available in Figure S8, Supplemental Digital Content, http://links.lww.com/MD/O86. Consistency was observed in the effect estimations of all SNPs for the HMGCR inhibition impact on DVT.

### 3.1. Sensitivity analysis

**Figure 5.**
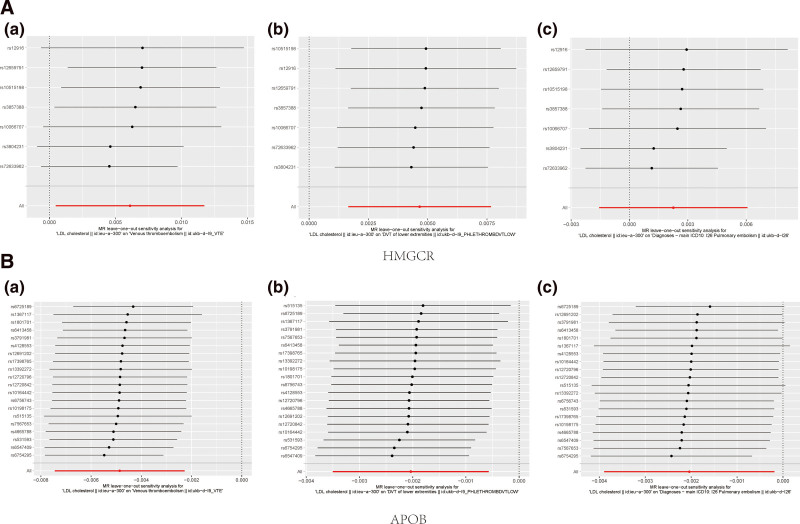
Leave-one-out analysis of genetically HMGCR and APOB inhibition on VTE, DVT and PE. (A): (a) HMGCR inhibitor on VTE risk; (b) HMGCR inhibitor on DVT risk; (c) HMGCR inhibitor on PE risk. (B) (a) APOB inhibitor on VTE risk; (b) APOB inhibitor on DVT risk; (c) APOB inhibitor on PE risk. APOB = apolipoprotein B, DVT = deep vein thrombosis, HMGCR = 3-hydroxy-3-methylglutaryl-CoA reductase, PE = pulmonary embolism, SNP = single nucleotide polymorphism, VTE = venous thromboembolism.

Tables S4–S6, Supplemental Digital Content, http://links.lww.com/MD/O85, http://links.lww.com/MD/O85, http://links.lww.com/MD/O85 presents the outcomes of MR-Egger, weighted median, Simple mode, and weighted mode analyses. Other MR methodologies yielded consistent results. No pleiotropic biases were apparent from the MR-Egger intercept test, enhancing credibility to the causal connection presented in Table S12, Supplemental Digital Content, http://links.lww.com/MD/O85. The figures delineated in both Figure 5 and Figure S9, Supplemental Digital Content, http://links.lww.com/MD/O86 display the leave-one-out analysis result, demonstrating that the causal impact evaluations associated with genetic proxies remain steady even when excluding any individual SNP from the HMGCR and APOB genes sets.

### 3.2. Colocalization

**Figure 6.**
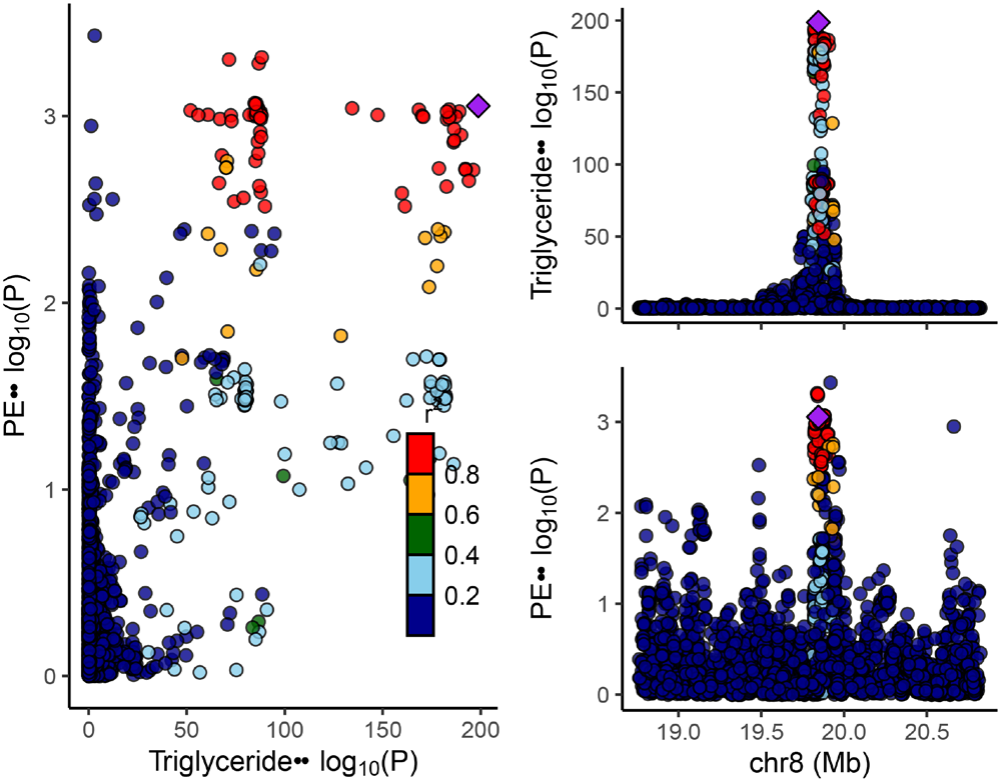
The colocalization visualization result for TG and PE within the LPL gene. LPL = lipoprotein lipase, PE = pulmonary embolism, TG = triglycerides.

For TG and PE, the likelihood of finding unique genetic variations within the LPL gene was remarkably smaller (0.31%) compared to the chance of identifying a shared causal variant (3.85%). The colocalization probability stood at 92.5% with a causal variant presumed present. The finding demonstrates that the effect of LPL on PE is not likely to be confounded by LD variants. Other colocalization results between gene targets that have a significant association with the outcome are presented in Table S13, Supplemental Digital Content, http://links.lww.com/MD/O85 and Figures S10–S12, Supplemental Digital Content, http://links.lww.com/MD/O86, http://links.lww.com/MD/O86, http://links.lww.com/MD/O86. The colocalization visualization result for TG and PE within the LPL gene, as an example, is shown in Figure 6. The *x*-axis depicts the genomic region, while the *y*-axis represents ‐log10(*P*) values. In the right portion, the upper panel illustrates TG data, while the lower panel displays outcome data. SNPs are denoted as points within the figure, color-coded according to significance level, where shades of red indicate greater significance.

## 4. Discussion

The genetic analysis conducted through drug-target MR has yielded several key findings within our study. We found that the inhibition of HMGCR is associated with a reduced likelihood of developing VTE and DVT, though our data did not support a protective effect against PE. Additionally, our results suggested a possible detrimental relationship between the inhibition of APOB and the activation of LPL with an increased risk of VTE and PE. Similarly, the inhibition of APOC3 and APOB may elevate the risk of DVT. It was worth mentioning that in our study, only LPL targeting substantially upped PE risk, with this observation verified through 2 strategies for creating genetic instruments based on TG-decreasing and Apo-B-decreasing variants in the LPL gene.

In MR studies targeting drug effects, colocalization analysis is frequently employed to ascertain the likelihood of colocalization. This is understood as the chance that a particular genetic variant impacts both the exposure and the outcome, in the context of an outcome-affecting causal variant. The colocalization probability is calculated as H4/(H3 + H4),^[26]^ signifying how a genetic variant simultaneously influences the exposure and outcome traits. The colocalization probability between TG-lowering genetic variants in LPL and PE was found to be 92.5%, indicating compelling evidence that both traits were influenced by the same genetic variation.

It is important to recognize that while certain findings yield statistical significance, the strength of these associations is modest, and statistical significance does not imply causation. The observed weak associations may be attributable to the intricate pathophysiology of VTE and the potential influence of unmeasured confounding variables. As the current research is grounded in population-level data, additional studies are necessary to validate the clinical relevance of these specific associations. While the observed associations in our study are modest, investigating the potential of lipid-lowering drugs to decrease the incidence of VTE and to lower associated mortality rates carries profound clinical relevance.

Traditionally, VTE and atherosclerotic cardiovascular diseases have been regarded as unrelated, characterized by their distinct clinical presentations and divergent pathophysiological pathways. However, emerging studies over recent years hint at a possible connection between venous and arterial thrombotic disorders.^[41,42]^ Statins, as representatives of lipid-lowering medications, lower lipid levels in the blood by blocking cholesterol production and facilitating the removal of low-density lipoprotein from the bloodstream, thereby serving to lower the occurrence or repetition of atherosclerotic incidents and ailments, including coronary heart disease, peripheral artery disease, and stroke.^[43,44]^ The benefits of statins may extend beyond their impact on lipid levels, encompassing their influence on thrombosis and inflammation.^[45,46]^ The hypothesized protective influence of statins on VTE has sparked significant interest within the research community. The pioneering evidence of statins’ preventive capability in VTE came with the Heart and Estrogen/Progestin Replacement Study results in 2002, where a comparative analysis showed statin users had half the VTE risk of nonusers.^[47]^ Subsequently, a host of observational studies and RCTs have investigated statins’ effectiveness for VTE primary prevention. Notably, the JUPITER trial, the inaugural RCT examining statins for VTE prevention, revealed that rosuvastatin lowered the risk of VTE by 45% and DVT by 55%. However, the reduction in PE risk did not reach statical significance.^[48]^ A meta-analysis undertaken by Kunutsor and colleagues, comprising 13 cohort studies and 23 RCTs, arrived at comparable findings.^[49]^

Recent scholarly inquiries have elucidated that inhibitors of PCSK9 also exhibit a propensity to diminish the incidence of VTE. In a detailed analysis following the FOURIER trial, Marston and colleagues investigated the potential of PCSK9 inhibitors to lower the incidence of VTE events. This investigation found that patients exhibiting higher initial levels of Lp(a) experienced a 33 nmol/L drop in these levels and a 48% reduction in VTE risk following evolocumab treatment. However, for individuals with lower initial Lp(a) levels, the reduction was a mere 7 nmol/L, and there was no perceptible reduction in their VTE risk.^[14]^ This suggests that lowering Lp(a) levels appears to significantly influence the reduction in VTE risk conferred by PCSK9 inhibitors. Nonetheless, there was no evidence indicating a VTE risk mitigation through PCSK9 gene suppression. In an additive component network meta-analysis, Farmakis^[50]^ evaluated the long-term VTE risk associated with various lipid-lowering therapy combinations, observing a dose-responsive trend where the summary effect size for VTE prevention enhanced with the intensification of lipid-lowering therapy. Importantly, the combination therapy of PCSK9 inhibitors and high-intensity statins emerged as a significantly more effective strategy for mitigating VTE risk than the use of low-to moderate-intensity statin monotherapy alone. In contrast, ezetimibe monotherapy did not affect the VTE risk. Moreover, our findings suggested that exposure to mipomersen directed at APOB might elevate VTE risk, including DVT and PE, Ibrolipim targeting LPL might increase the risk of VTE and PE, and Volanesorsen targeting APOC3 also showed a similar phenomenon in DVT, but these points have not yet been confirmed. Since these drugs have been approved by regulatory authorities in multiple countries, evaluating their VTE risk effects relative to other lipid-lowering medications presents a viable comparison opportunity.

Various mechanisms are proposed to account for the protective action of statins against VTE. Statins have been shown to cause a marked decrease in the blood coagulation cascade, likely through diminished tissue factor expression, resulting in decreased thrombin production.^[45]^ Statins enhance endothelial function by stimulating and upregulating endothelial NO synthase, leading to increased endothelial NO production, and by exerting antioxidant effects.^[51]^ They also boost Kruppel-like factor-2 activity, enhance the expression of thrombomodulin, and reduce PAI-1 expression in human endothelial cells.^[52]^ Moreover, statins prevent the isoprenylation of signaling proteins, offering potential antithrombotic effects by diminishing fibrinogen cleavage and the activation of factors V and VIII.^[53]^ Furthermore, the direct anti-inflammatory properties of statins are thought to help lower the occurrence of VTE.^[54]^ Although these pathways suggest how inhibition of HMGCR might lower VTE risks, further investigation is required to fully understand and confirm the roles and interactions of these mechanisms.

Extensive research and biological mechanisms indicating a positive impact of statin therapy on the risk of VTE, but there remains some inconsistency in study findings. Rahimi and colleagues, in a meta-analysis of twenty-two studies comparing statins with a control group across 105,759 individuals and 7 studies on high versus standard dose statins among 40,594 people, found no significant decrease in VTE risk associated with statin therapy.^[55]^ A study involving 32,062 patients with VTE found that the recurrence rate of VTE among statin users was comparable to that of nonusers. However, statin users experienced a higher incidence of major bleeding, while mortality rates were similar between the 2 groups. After adjusting for confounding variables through propensity score matching, the risk of VTE recurrence and major bleeding in statin users remained analogous to that of nonusers.^[56]^ Smeeth and colleagues conducted a population-based cohort study to assess the impact of statins on various health outcomes, revealing limited evidence to support a reduction in the risk of VTE associated with statin use.^[57]^ In addition, a network meta-analysis, along with an additional comparative study, indicated that fenofibrate was associated with an increased incidence of VTE events.^[58,59]^ The protective effect of statins against VTE remains a topic of debate, highlighting the need for further high-quality studies to establish whether statin therapy can effectively reduce the risk of recurrent VTE and associated mortality.

The primary strength of this study lay in its thorough examination of the targets of both established and emerging lipid-lowering medications, encompassing statins, evolocumab/alirocumab, ezetimibe, mipomersen, fibrates, Ibrolipim, evinacumab, and volanesorsen. Furthermore, a range of sensitivity analyses were conducted to assess the robustness of the findings against underlying presuppositions or multi-factorial influences. Nevertheless, while analyzing these results, recognizing some limitations is crucial. Firstly, this study focused specifically on the expected effects of drug targets without estimating potential non-intended nontarget effects. Given that drugs often elicit a slew of effects beyond their primary targets, this aspect warrants consideration in the analysis. Secondly, the MR approach used relies on assumptions about the instrumental variables that cannot be directly proven, and there could be multivariable interference or confounders skewing these estimates, despite the reassurance provided by sensitivity analyses. Thirdly, the colocalization analysis performed in this study suggests a low likelihood of shared causal variants, possibly reflecting limited power or the non-presence of causal variants in the exposure and outcome genetic data. Finally, the concentration on individuals with European ancestry highlights a need for further research to ascertain the findings’ universal applicability, emphasizing the value of extending these studies to more racially and ethnically diverse groups.

## 5. Conclusions

In this study, we investigated the genetic correlation between lipid-lowering drug-target genes and the incidence of VTE. Our findings indicate that HMGCR inhibition is associated with a decreased risk of developing VTE and deep vein thrombosis, although no protective effect was observed concerning pulmonary embolism. Conversely, we did not find any significant impact of 3 lipid-lowering agents—ezetimibe, PCSK9 inhibitors, and angiopoietin-like 3 inhibitors—on the incidence of VTE, DVT, or PE. These results highlight the need for further research to clarify the underlying mechanisms involved.

## Acknowledgments

We thank all the authors and participants of the GWAS.

## Author contributions

**Data curation:** Yu Tan, Min Liu.

**Investigation:** Min Li, Hangyu Duan.

**Methodology:** Min Li, Hangyu Duan, Xiaohan Zhao.

**Software:** Jinwen Luo.

**Writing – original draft:** Min Li, Hangyu Duan.

**Writing – review & editing:** Dazhuo Shi, Xiaojuan Ma.

## Supplementary Material


