# Supplementary material for: Genetic correlation between genes targeted by lipid-lowering drugs and venous thromboembolism: A drug-target Mendelian randomization study
Source: Medicine (Baltimore). 2024 Dec 20;103(51):e40770. doi: 10.1097/MD.0000000000040770 (PMC11666180; doi:10.1097/MD.0000000000040770)
Supplement: Supplementary file 2 [file medi-103-e40770-s002.pdf]

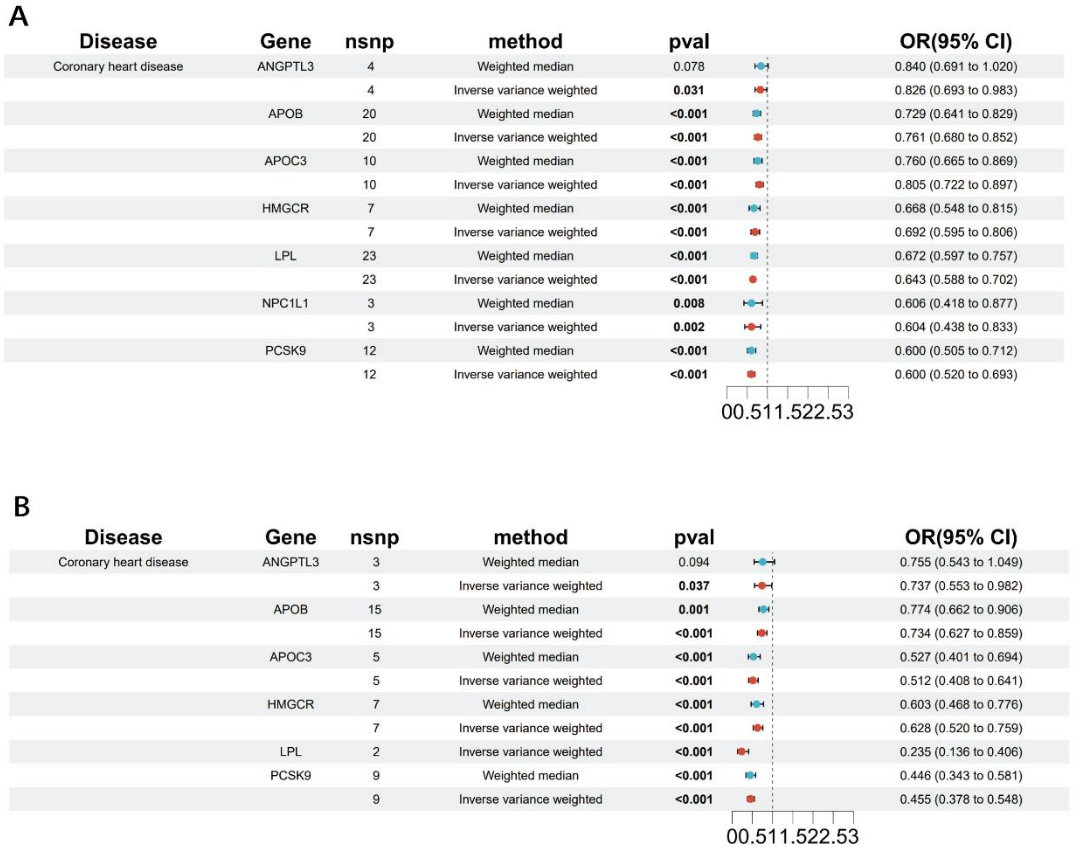

Figure S1. Association of genetically-proxied drug targets with risk of coronary heart disease. Forest plot A is the association of genetically proxied lipid-lowering drug targets with risk of CHD using primary lipid modifying effect. Forest plot B is the association of genetically proxied lipid-lowering drug targets with risk of CHD using alternative lipid modifying effect. Data are represented as odds ratios (ORs) with 95% confidence intervals (error bars). An OR of <1.00 suggests a decreased risk of disease associated with lipid-lowering drug treatment. Abbreviations: CHD, Coronary heart disease; OR, odds ratio; Apo-B, Apolipoprotein-B; SNP, single-nucleotide polymorphisms; HMGCR, HMG-CoA reductase; NPC1L1, Niemann-Pick C1-like protein 1; PCSK9, proprotein convertase subtilisin/kexin type 9; APOB, Apolipoprotein B-100; ANGPTL3, angiopoietin-like 3; APOC3, Apolipoprotein C-III; LPL, lipoprotein lipase.

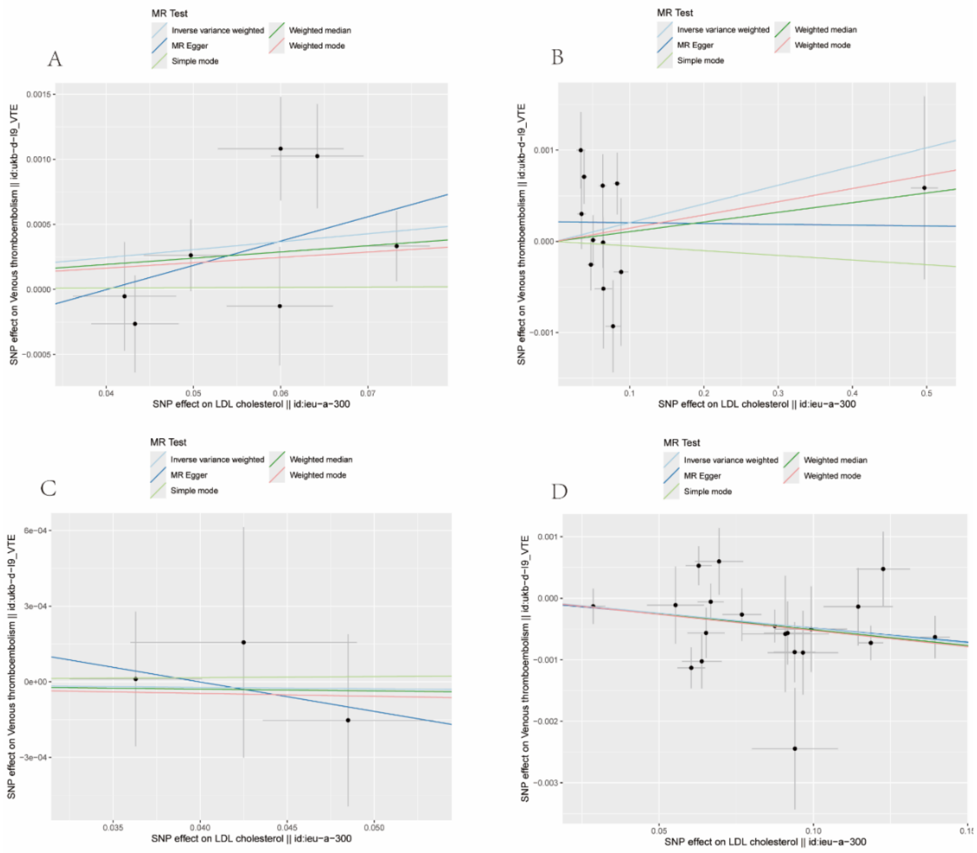

Figure S2. Scatter plot of the association between LDL and VTE using SNPs within or near the HMGCR, PCSK9, NPC1L1, and APOB locus.

A. HMGCR inhibitor on VTE risk; B. PCSK9 inhibitor on VTE risk; C. NPC1L1 inhibitor on VTE risk. D. APOB inhibitor on VTE risk.

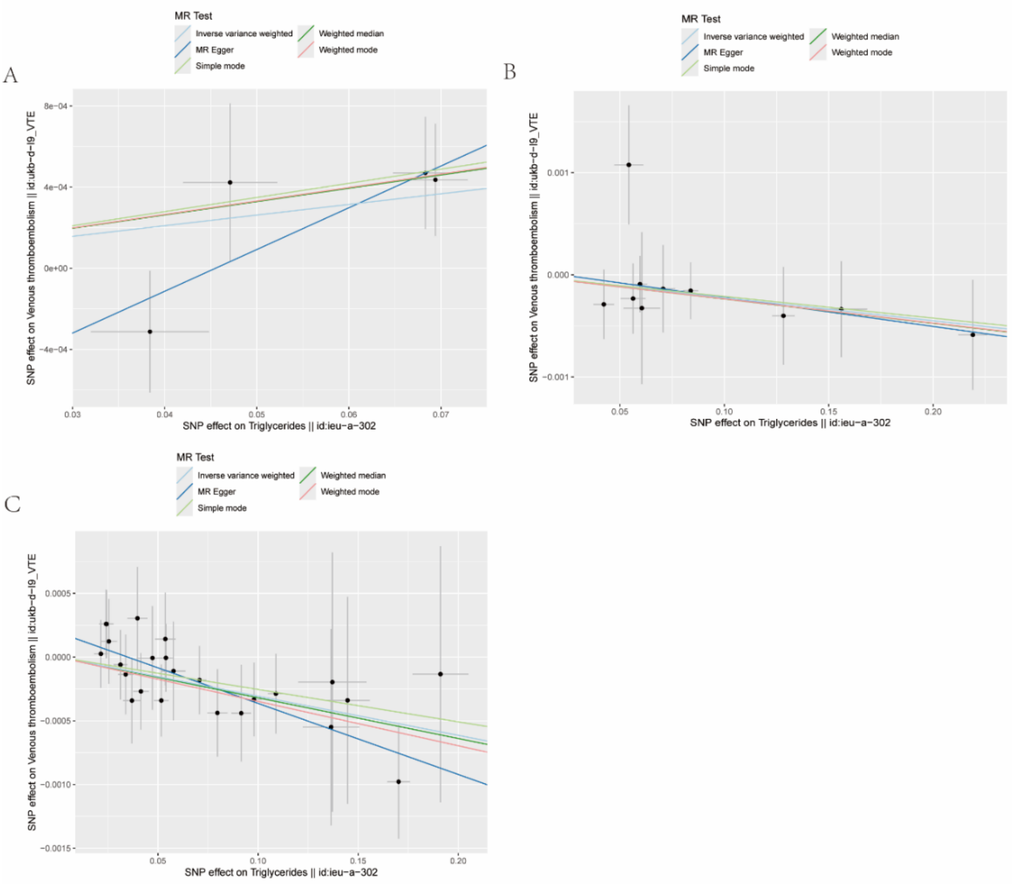

Figure S3. Scatter plot of the association between TG and VTE using SNPs within or near the ANGPTL3, APOC3 and LPL locus.

A. ANGPTL3 inhibitor on VTE risk; B. APOC3 inhibitor on VTE risk; C. LPL inhibitor on VTE risk.

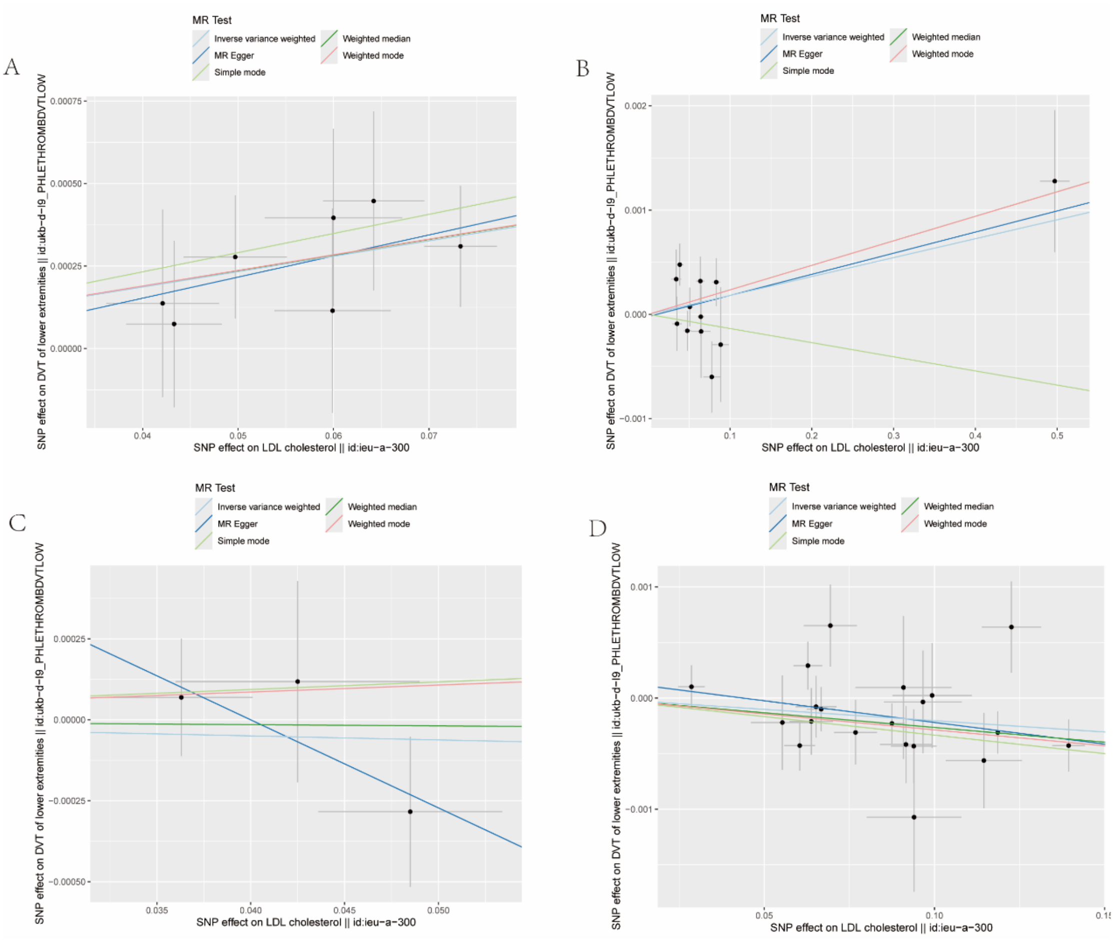

Figure S4. Scatter plot of the association between LDL and DVT using SNPs within or near the HMGCR, PCSK9, NPC1L1, and APOB locus.

A. HMGCR inhibitor on DVT risk; B. PCSK9 inhibitor on DVT risk; C. NPC1L1 inhibitor on DVT risk. D. APOB inhibitor on DVT risk.

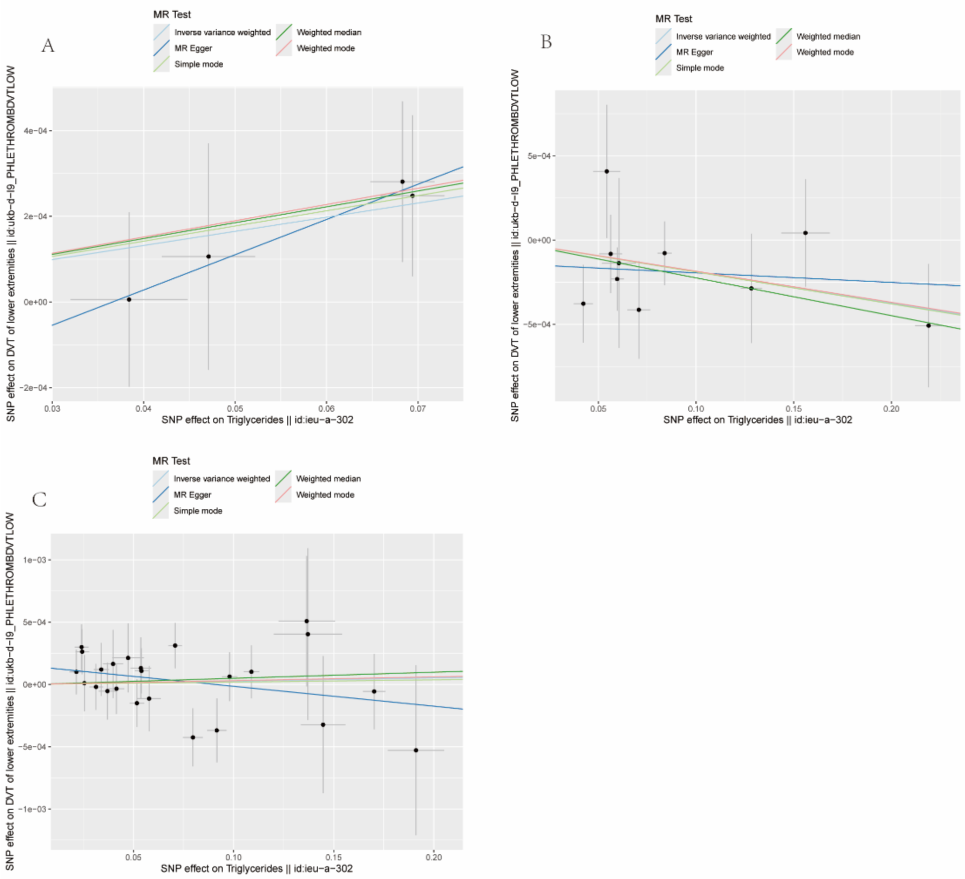

Figure S5. Scatter plot of the association between TG and DVT using SNPs within or near the ANGPTL3, APOC3 and LPL locus.

A. ANGPTL3 inhibitor on DVT risk; B. APOC3 inhibitor on DVT risk; C. LPL inhibitor on DVT risk.

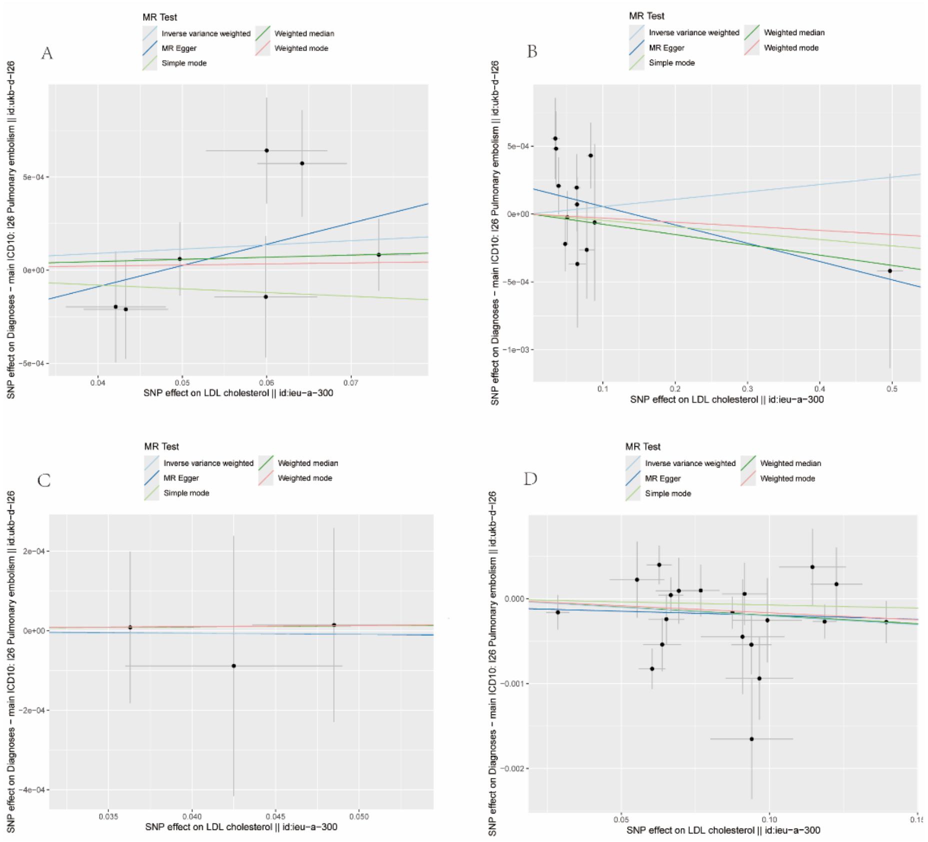

Figure S6. Scatter plot of the association between LDL and PE using SNPs within or near the HMGCR, PCSK9, NPC1L1, and APOB locus.

A. HMGCR inhibitor on PE risk; B. PCSK9 inhibitor on PE risk; C. NPC1L1 inhibitor on PE risk. D. APOB inhibitor on PE risk.

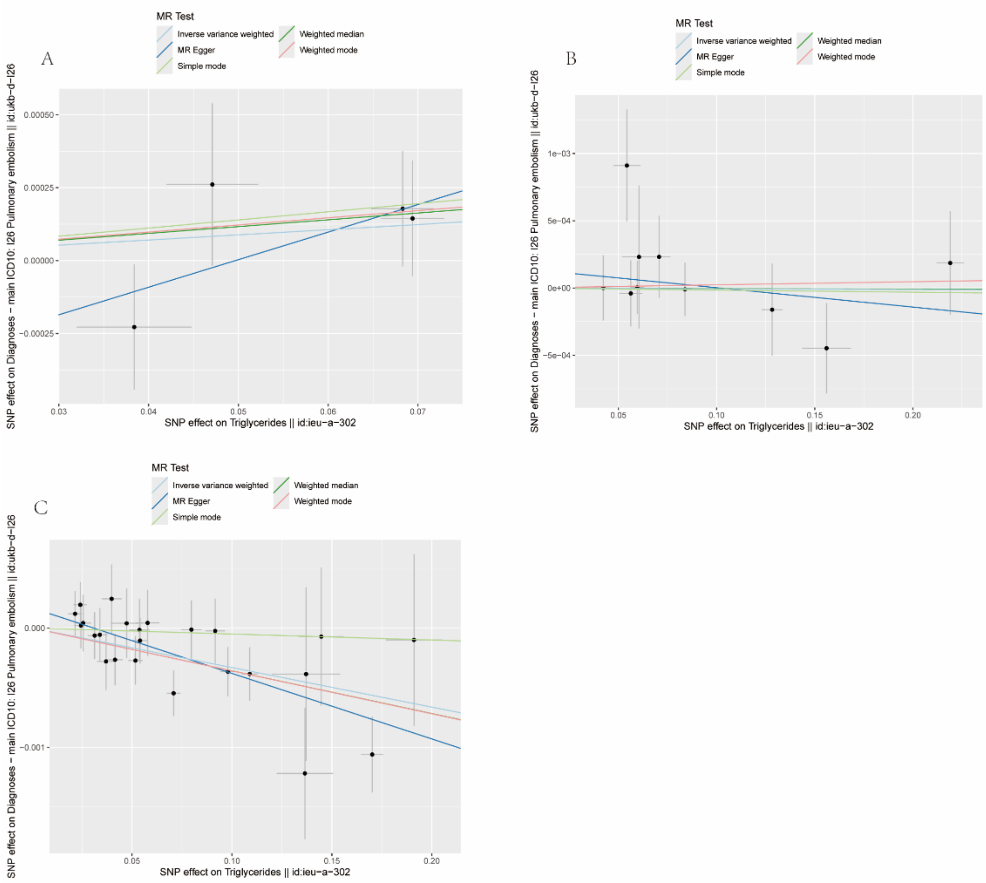

Figure S7. Scatter plot of the association between TG and PE using SNPs within or near the ANGPTL3, APOC3 and LPL locus.

A. ANGPTL3 inhibitor on PE risk; B. APOC3 inhibitor on PE risk; C. LPL inhibitor on PE risk.

A

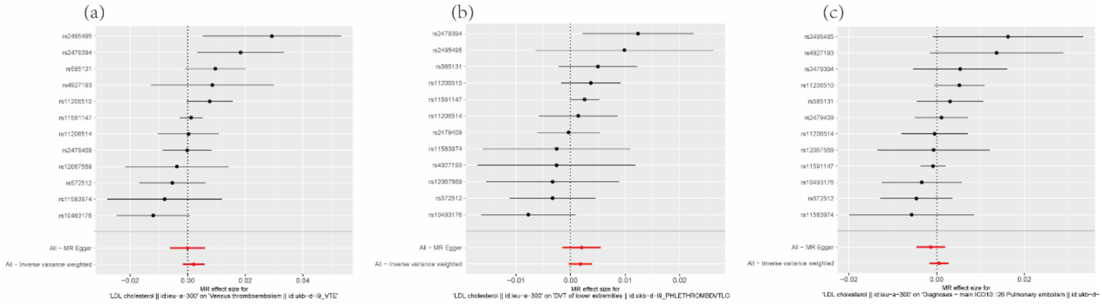

PSCK9

B

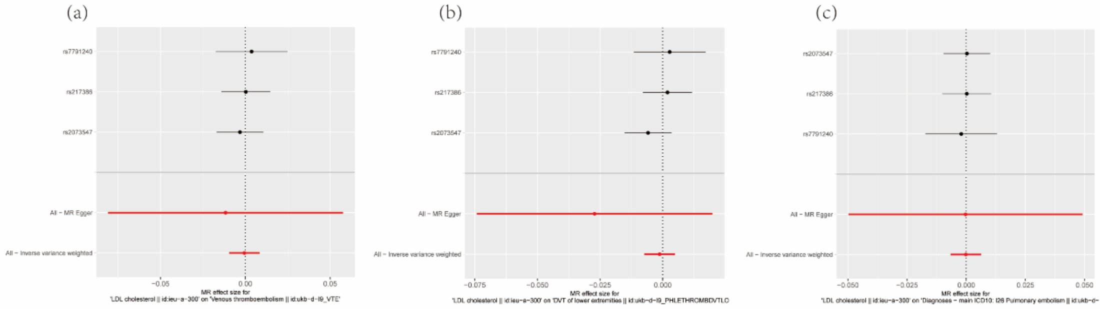

NPC1L1

C

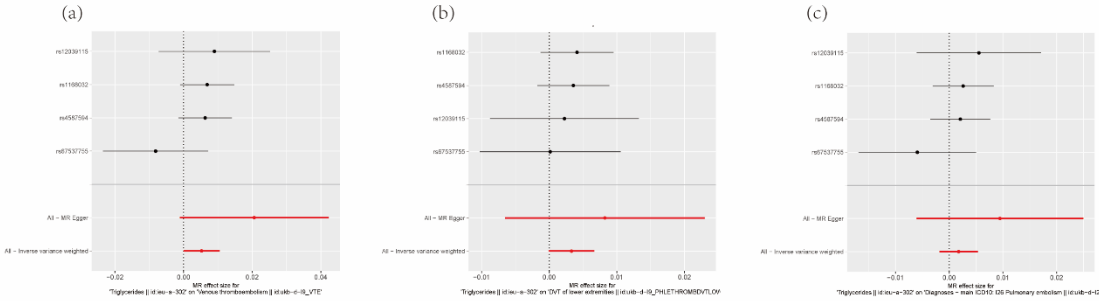

ANGPTL3

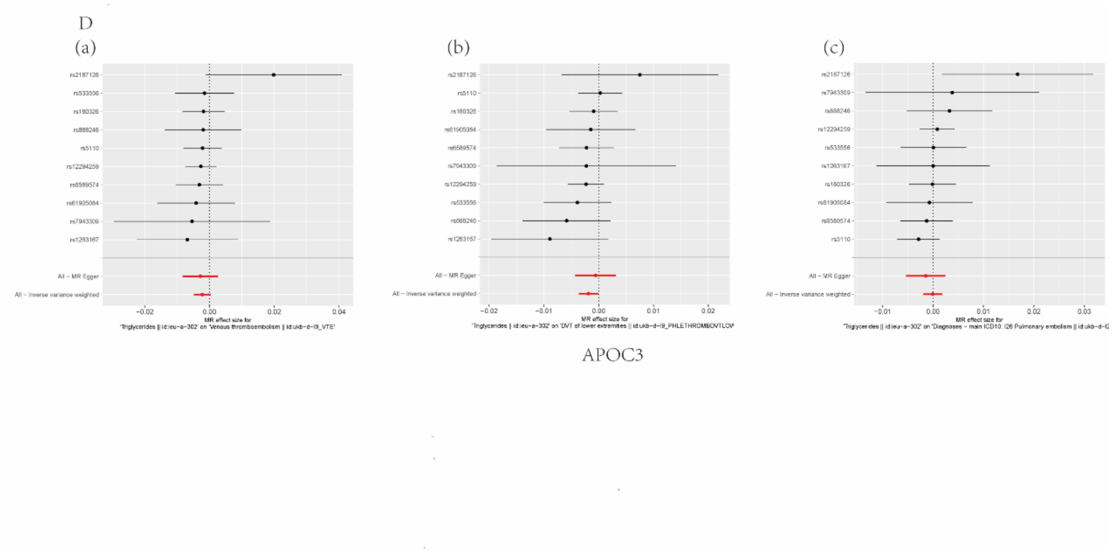

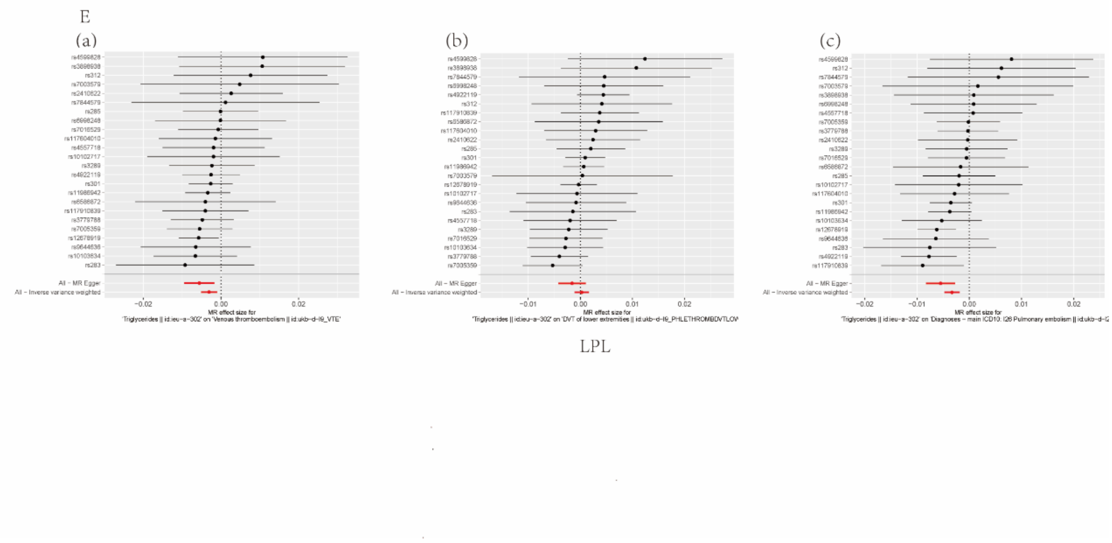

Figure S8. Forest plots showed the casual effect estimates of each SNP in PCSK9, NPC1L1, ANGPTL3, APOC3 inhibition and LPL activation instruments on VTE, DVT and PE.

A. (a) PCSK9 inhibitor on VTE risk; (b) PCSK9 inhibitor on DVT risk; (c) PCSK9 inhibitor on PE risk. B. (a) NPC1L1 inhibitor on VTE risk; (b) NPC1L1 inhibitor on DVT risk; (c) NPC1L1 inhibitor on PE risk. C. (a) ANGPTL3 inhibitor on VTE risk; (b) ANGPTL3 inhibitor on DVT risk; (c) ANGPTL3 inhibitor on PE risk. D. (a) APOC3 inhibitor on VTE risk; (b) APOC3 inhibitor on DVT risk; (c) APOC3 inhibitor on PE risk. E. (a) LPL activation on VTE risk; (b) LPL activation on DVT risk; (c) LPL activation on PE risk.

A

(a) (b) (c)

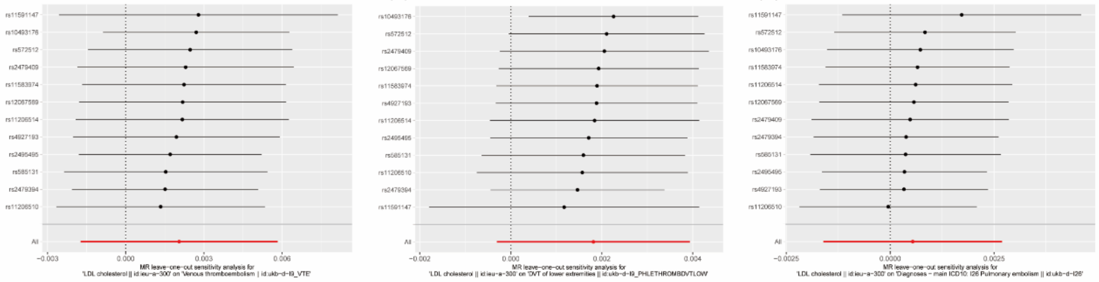

PCSK9

B

(a) (b) (c)

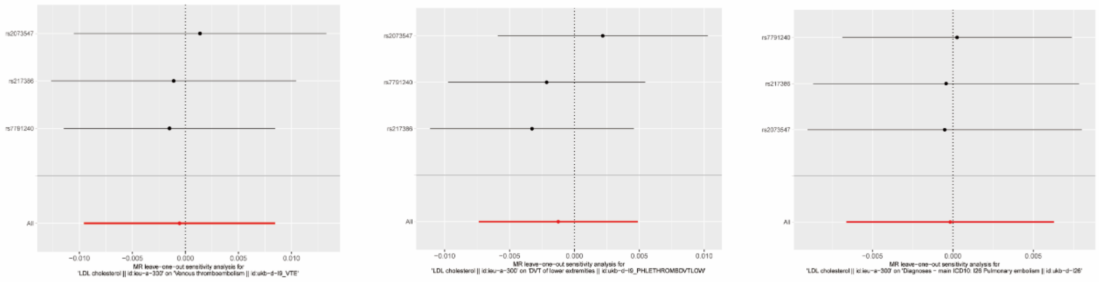

NPC1L1

C

(a) (b) (c)

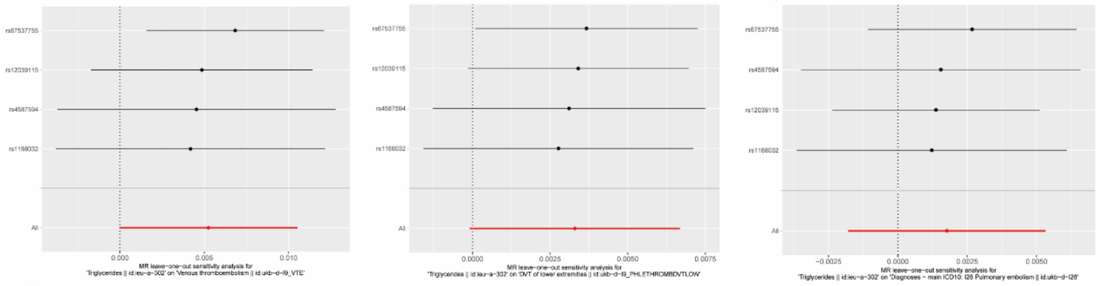

ANGPTL3

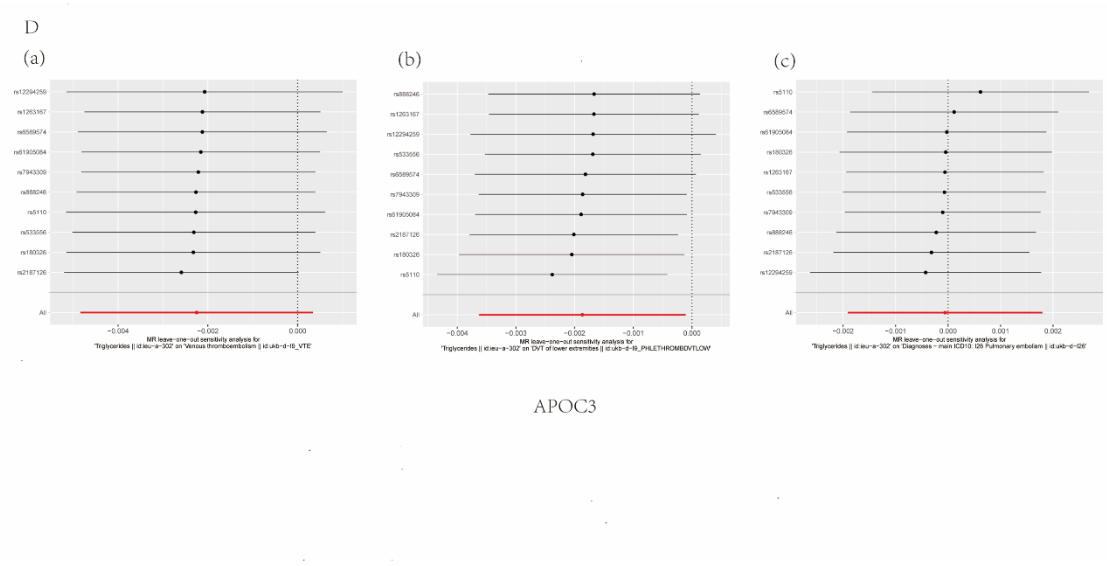

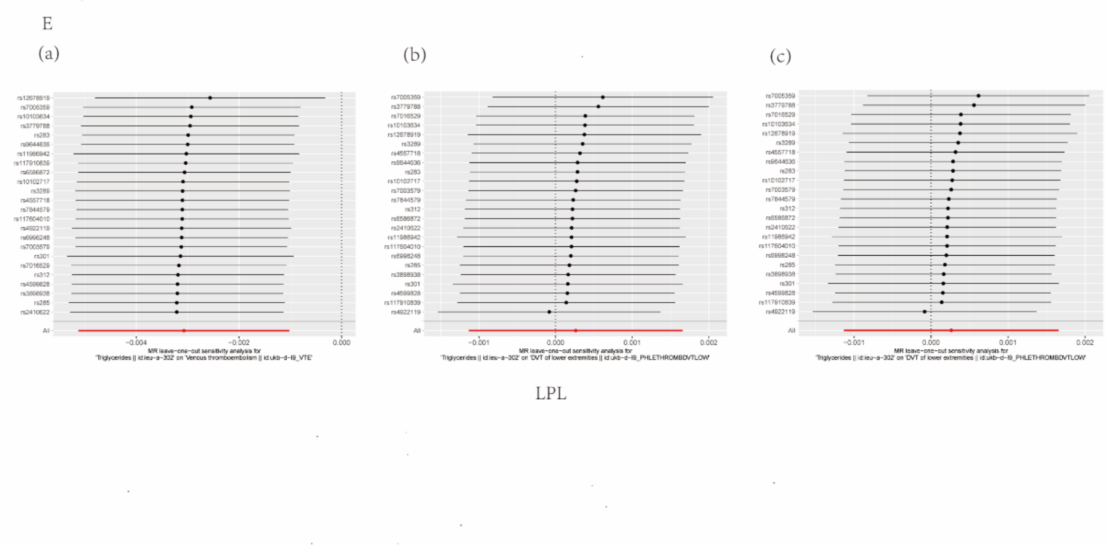

Figure S9. Leave-one-out analysis of genetically PCSK9, NPC1L1, ANGPTL3, APOC3 inhibition and LPL activation instruments on VTE, DVT and PE.

A. (a) PCSK9 inhibitor on VTE risk; (b) PCSK9 inhibitor on DVT risk; (c) PCSK9 inhibitor on PE risk. B. (a) NPC1L1 inhibitor on VTE risk; (b) NPC1L1 inhibitor on DVT risk; (c) NPC1L1 inhibitor on PE risk. C. (a) ANGPTL3 inhibitor on VTE risk; (b) ANGPTL3 inhibitor on DVT risk; (c) ANGPTL3 inhibitor on PE risk. D. (a) APOC3 inhibitor on VTE risk; (b) APOC3 inhibitor on DVT risk; (c) APOC3 inhibitor on PE risk. E. (a) LPL activation on VTE risk; (b) LPL activation on DVT risk; (c) LPL activation on PE risk.

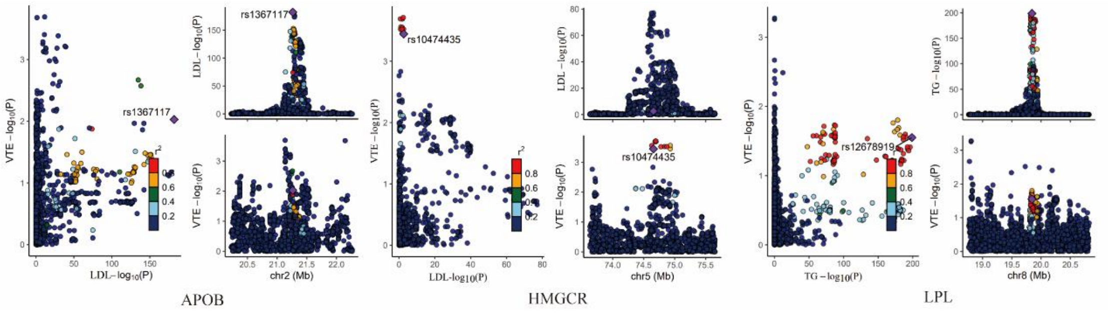

Figure S10. The colocalization visualization result for VTE within the APOB, HMGCR and LPL gene.

A. The colocalization visualization result for LDL-C and VTE within the APOB gene. B. The colocalization visualization result for LDL-C and VTE within the HMGCR gene. C. The colocalization visualization result for TG and VTE within the LPL gene.

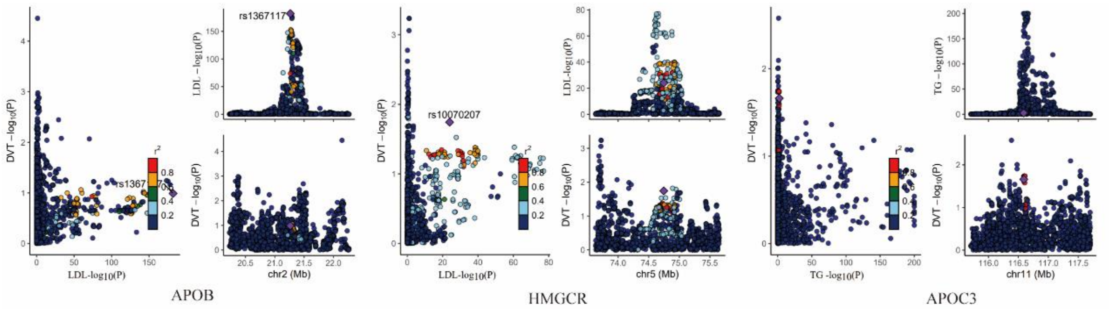

Figure S11. The colocalization visualization result for DVT within the APOB, HMGCR and APOC3 gene.

A. The colocalization visualization result for LDL-C and DVT within the APOB gene. B. The colocalization visualization result for LDL-C and DVT within the HMGCR gene. C. The colocalization visualization result for TG and VTE within the APOC3 gene.

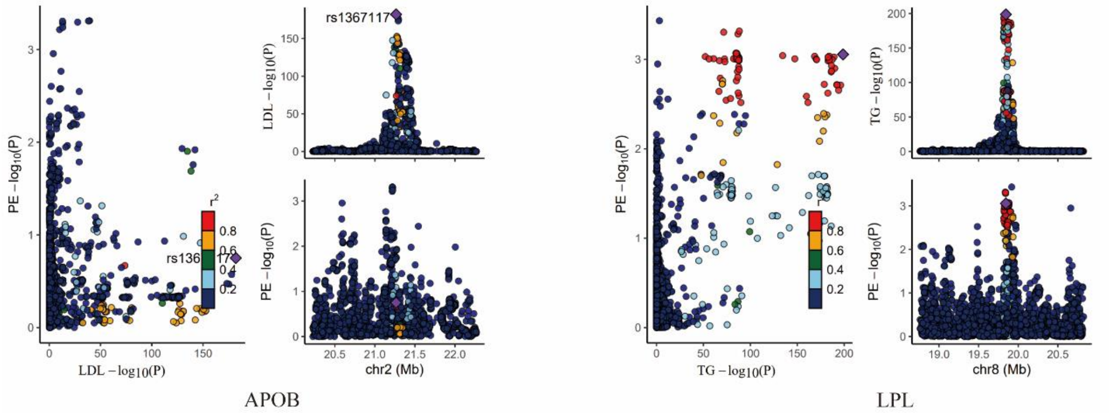

Figure S12. The colocalization visualization result for PE within the APOB and LPL gene.

A. The colocalization visualization result for LDL-C and PE within the APOB gene. B. The colocalization visualization result for TG and PE within the LPL gene.
